# Determination of candidate metabolite biomarkers associated with recurrence of HCV-related hepatocellular carcinoma

**DOI:** 10.18632/oncotarget.23500

**Published:** 2017-12-15

**Authors:** Zhicheng Liu, Pierre Nahon, Zaifang Li, Peiyuan Yin, Yanli Li, Roland Amathieu, Nathalie Ganne-Carrié, Marianne Ziol, Nicolas Sellier, Olivier Seror, Laurence Le Moyec, Philippe Savarin, Guowang Xu

**Affiliations:** ^1^ CAS Key Laboratory of Separation Science for Analytical Chemistry, Dalian Institute of Chemical Physics, Chinese Academy of Sciences, Dalian, China; ^2^ Université Paris 13, Sorbonne Paris Cité, Laboratoire de Chimie, Structures et Propriétés de Biomateriaux et d’Agents Therapeutiques, UMR 7244, Bobigny, France; ^3^ Hepatology Unit, Jean Verdier Teaching Hospital, AP-HP, Bondy, France; ^4^ INSERM U1162, Génomique Fonctionnelle des Tumeurs Solides, INSERM U1162, Paris, France; ^5^ Intensive Care Unit, Jean Verdier Teaching Hospital, AP-HP, Bondy, France; ^6^ APHP, Service d'Anatomie Pathologique, Hôpital Jean Verdier, BB-0033-00027, Centre de Ressources Biologiques Maladies du foie, Groupe Hospitalier, Paris-Seine-Saint-Denis, France; ^7^ BB-0033-00027, Centre de Ressources Biologiques Maladies du Foie, Groupe Hospitalier Paris-Seine-Saint-Denis, Bondy, France; ^8^ APHP, Service de Radiologie, Hôpital Jean Verdier, Bondy, France; ^9^ Université d’Evry Val d’Essonne, UBIAE, EA7362, Evry, France; ^10^ University Paris 13, Bobigny, France

**Keywords:** HCV-related HCC, recurrence, GC-MS, metabolomics, metabolic biomarker

## Abstract

Hepatitis C virus (HCV) infection is associated with a high risk of developing hepatocellular carcinoma (HCC) and HCC recurrence remains the primary threat to outcomes after curative therapy. In this study, we compared recurrent and non-recurrent HCC patients treated with radiofrequency ablation (RFA) in order to identify characteristic metabolic profile variations associated with HCC recurrence. Gas chromatography-mass spectrometry (GC-MS) -based metabolomic analyses were conducted on serum samples obtained before and after RFA therapy. Significant variations were observed in metabolites in the glycerolipid, tricarboxylic acid (TCA) cycle, fatty acid, and amino acid pathways between recurrent and non-recurrent patients. Observed differences in metabolites associated with recurrence did not coincide before and after treatment except for fatty acids. Based on the comparison of serum metabolomes between recurrent and non-recurrent patients, key discriminatory metabolites were defined by a random forest (RF) test. Two combinations of these metabolites before and after RFA treatment showed outstanding performance in predicting HCV-related HCC recurrence, they were further confirmed by an external validation set. Our study showed that the determined combination of metabolites may be potential biomarkers for the prediction of HCC recurrence before and after RFA treatment.

## INTRODUCTION

Hepatocellular carcinoma (HCC) is one of the most common cancers in the world [1]. Despite improvements in survival rates in recent years [2, 3], its worldwide incidence and malignancy-associated death rates are still challenging due to high rates of recurrence and difficulties associated with prognosis assessment. Among curative strategies, radiofrequency ablation (RFA) is preferred when HCC is diagnosed at an early stage or when the tumor size is small. This approach has demonstrated safety and relative simplicity, and creates a minimal operative wound for patients [4].

The worldwide prevalence of hepatitis C-related HCC has been increasing in recent years. In particular, in countries such as Japan, Egypt, and some African countries, hepatitis C virus (HCV) infection is the primary cause of HCC [5]. It has been suggested that HCV contributes to HCC by directly modulating pathways that promote malignant transformation of hepatocytes [6]. HCC may be induced indirectly via HCV-associated chronic inflammation, cell death, proliferation, and cirrhosis [7].

Beyond the traditional marker, alpha fetoprotein (AFP), various new biomarkers have been applied during clinical assessment as tools for early screening and prediction of outcomes after the implementation of curative procedures [8]. Metabolomics has been used in the early diagnosis and prognosis of various cancers, including HCC [9, 10]. Our previous metabolomic studies have revealed differences in the metabolic profiles of HCC patients compared to healthy controls or cirrhotic patients [11–13], and liquid chromatography-mass spectrometry (LC-MS) can be used to discriminate HCC early recurrence from late recurrence [14]. Similar studies have also been reported by other researchers [15–20]. However, in most of these previous HCC studies, the distinction between development of HCC in hepatitis virus-infected patients versus non-viral patients was not taken into consideration. In one of our previous studies, using nuclear magnetic resonance (NMR)-based metabolomics, we demonstrated that metabolic profiles from viral HCC patients are distinct from those of non-viral HCC patients [21]. It is well known that gas chromatography-mass spectrometry (GC-MS) is more sensitive than NMR. In the present study, we used GC-MS-based metabolomics in order to investigate changes in serum metabolic profiles before and after RFA implementation in a homogeneous cohort of HCV-related HCC patients in order to identify potential metabolite candidate biomarkers associated with risk of HCC tumor recurrence.

## RESULTS

For the samples in the training set, data pretreatment was started with 16 quality control (QC) samples. After screening ion peak areas on the basis of the acquired data from the QC samples, (shown in Supplementary Information 1), 230 peaks corresponding to compounds in the serum were obtained. Peaks in the test (non-control) samples were identified qualitatively according to the peaks characterized in the QC samples and then quantified by integration of their areas. The recorded peak area represents the relative concentration of the corresponding compound in the serum. A data set for test samples containing peak area information for all 230 defined peaks was established and analyzed in the following discriminatory analyses. An assessment by principal component analysis (PCA) for all QC samples was performed (Supplementary Figure 1). Focalization of the QC samples was observed by comparing to all other test samples, and showed good stability of the analytical sequence.

For the experiments with the samples in the validation set, the relative standard deviation (RSD) of the targeted peaks in the 9 QC samples was obtained, shown in Supplementary Table 1. L-norvaline, with the lowest RSD area, was chosen as the normalization reference to calculate the relative area of each targeted ion (Supplementary Table 1). For all metabolites that corresponded to 2 or more ion peaks, the peak with the smallest RSD was selected and submitted for receiver operating curve (ROC) tests.

### Baseline characteristics of patients

**Figure 1:**
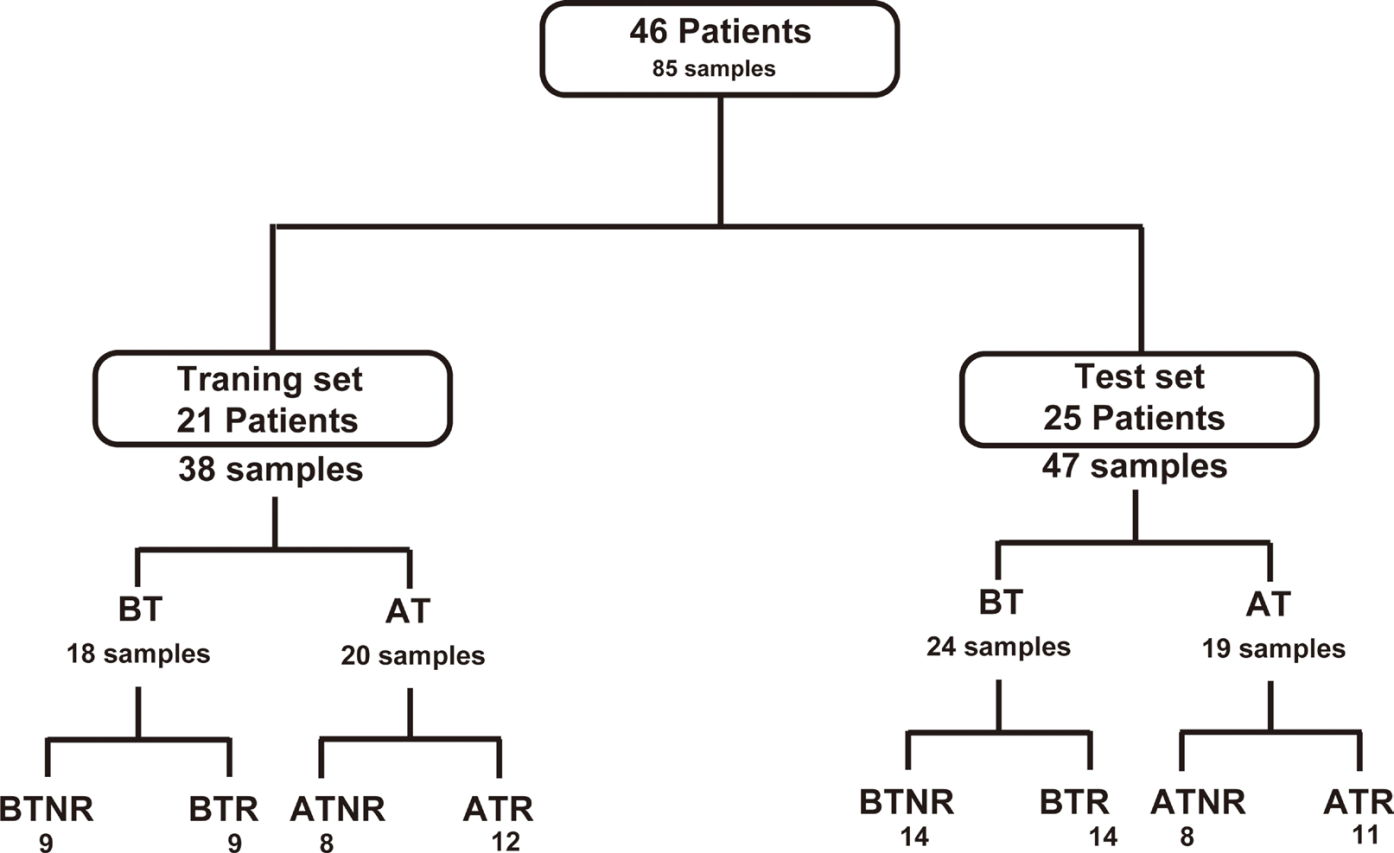
Flowchart of the distribution of patients and samples BT: before RFA therapy, AT: after RFA therapy; NR: HCC patients without recurrence; R: patients with recurrence.

Eighty-five samples from 46 patients fulfilling the inclusion criteria were included in the study. Among the samples, 38 were included in the training set while the remaining 47 were included in the validation set. Design of the current study is depicted in Figure 1. The samples were grouped by sampling time (BT: before RFA therapy; AT: 2 months after RFA therapy) and recurrence status (NR: non-recurrent patients; R: recurrent patients). Baseline information for all included patients is shown in Table 1. On the basis of the statistical data in the table, no significant variation (*p* > 0.05) from the clinical assessments was found between the recurrent group and the non-recurrent group, both in the training set and validation set samples.

**Table 1:** Baseline characteristics of the enrolled patients in the study

	Training Set				Validation set			
	Total Training set	Patients with HCC recurrence	Patients without HCC recurrence	*P*-value_Rec_	Total Validation set	Patients with HCC recurrence	Patients without HCC recurrence	*P*-value_Rec_
**Number of patients**	21	11 (52%)	10 (45%)	NA	25	13 (52%)	12 (48%)	NA
**Age (Average)**	70.6 ± 0.3	70.2 ± 0.4	69.7 ± 0.3	0.22	67.6 ± 0.5	65.8 ± 0.9	69.4 ± 1.0	0.23
**Gender (Male %)**	18 (86%)	9 (82%)	9 (90%)	NA	17 (68%)	8 (61%)	9 (75%)	NA
**Uni-nodular HCC**	16 (76%)	9 (81%)	7 (70%)	NA	20 (80%)	10 (77%)	10 (83%)	NA
**Largest nodule size (mm)**	28.2 ± 0.2	28 ± 0.4	28.4 ± 0.4	0.44	23.6 ± 0.3	26.1 ± 0.6	21.0 ± 0.7	0.06
**AFP (ng/ml)**	44.4 ± 2.7	52.6 ± 5.5	36.5 ± 5.3	0.34	28.9 ± 1.6	38.7 ± 3.3	19.1 ± 2.8	0.10
**TG (g/L)**	1.1 ± 0.02	1.1 ± 0.03	1.1 ± 0.07	0.47	1.0 ± 0.02	1.0 ± 0.05	0.9 ± 0.03	0.39
**Cholesterol (g/L)**	4.4 ± 0.03	4.5 ± 0.05	4.2 ± 0.07	0.20	3.8 ± 0.07	3.5 ± 0.07	4.1 ± 0.16	0.14
**Glycemia (IU/L)**	6.6 ± 0.32	6.9 ± 0.08	6.4 ± 0.06	0.18	6.5 ± 0.07	6.3 ± 0.12	6.6 ± 0.17	0.33
**AST (IU/L)**	58.4 ± 1.1	64.3 ± 3.1	53 ± 0.9	0.26	56.4 ± 1.2	62.8 ± 2.6	50.1 ± 2.3	0.15
**ALT (IU/L)**	49.5 ± 0.7	50.3 ± 1.7	48.8 ± 1.1	0.45	45.6 ± 1.2	48.8 ± 2.7	42.5 ± 2.3	0.31
**GGT (IU/L)**	123.2 ± 1.9	140.0 ± 4.7	107.7 ± 3.5	0.13	108.2 ± 4.6	110.7 ± 4.4	105.6 ± 12.6	0.46

All the percentages were calculated as a proportion of all enrolled patients. Uni-nodular: HCC patients who had only one nodule of tumor. AFP: alpha fetoprotein; TG: triglyceride; AST: aspartate aminotransferase; ALT: alanine aminotransferase; GGT: γ-glutamyl transpeptidase. *P*-value_Rec_: *P*-value for the comparison between recurrent and non-recurrent HCC patients. The tolerance scope was calculated by the standard error (SE).

### Discriminatory analyses according to HCC recurrence status

**Figure 2:**
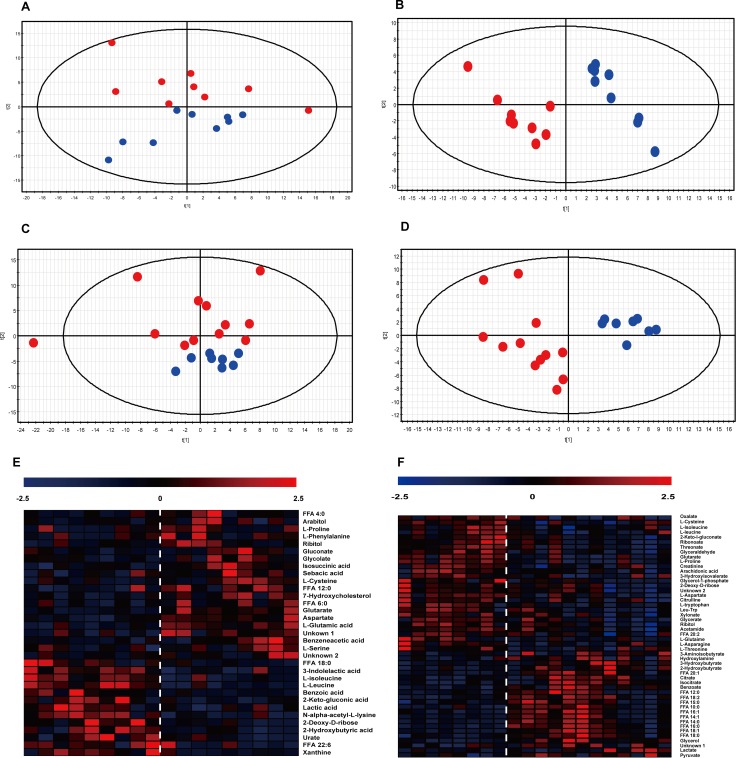
Multivariate analyses for RBT vs. NRBT. and RAT vs. RBT (**A**–**D**) Score-plot of PCA and PLS-DA. The abscissa of the score-plots represents the contribution of the first component and the ordinate stands for the contribution of the second component of the analysis. Blue dots: non-recurrent patients; red dots: recurrent patients. A: PCA for RBT and NRBT samples. B: PLS-DA for the separation between NRBT and RBT. C: PCA of RAT and NRAT samples. D: PLS-DA for the separation between RAT and NRAT. (**E**–**F**) Heat map presenting the hierarchical clustering analysis for the two separations, to the left of the dotted line: non-recurrent patients; to the right of the dotted line: recurrent patients. Each column represnets a sample while each row represents a significantly varied metabolite. The color is corresponding to the fold change of the metabolite. E: heat map for RBTs vs. NRBTs; F: heat map for RAT vs. NRAT.

Eighteen samples were collected before RFA treatment while 20 samples collected 2 months after RFA were available. PCA and partial least squares discriminant analysis (PLS-DA) were performed for the two cohorts, shown in Figure 2. According to the multivariate analyses, clear differences between R_BT_ vs. NR_BT_ (Figure 2A, 2B) and R_AT_ vs. NR_AT_ (Figure 2C–2D) were observed. Significant differences were observed between patients with and without recurrence, before and after RFA treatment. With 2 components, the Q^2^ which indicates predictability of the discriminant model, values for the two models were 0.34 and 0.5 for the before treatment model and after treatment model, respectively. This result exhibited that the discriminatory models predicted well the differences between the recurrent and non-recurrent patients before and after RFA treatment. Cross validation for the two models demonstrated that they were not over-fitting (shown in Supplementary Figure 2). According to the PLS-DA analyses, the contribution of each metabolite to the model was determined by the variable importance in the projection (VIP). Metabolites with a VIP superior to 1 for the two models, along with their *P*-values and fold-change, are listed in Supplementary Table 2. Heat maps showing the different variation directions and fold-changes for each sample are presented in Figure 2E–2F.

### Metabolomic differences before RFA therapy (BT) according to subsequent HCC recurrence

**Figure 3:**
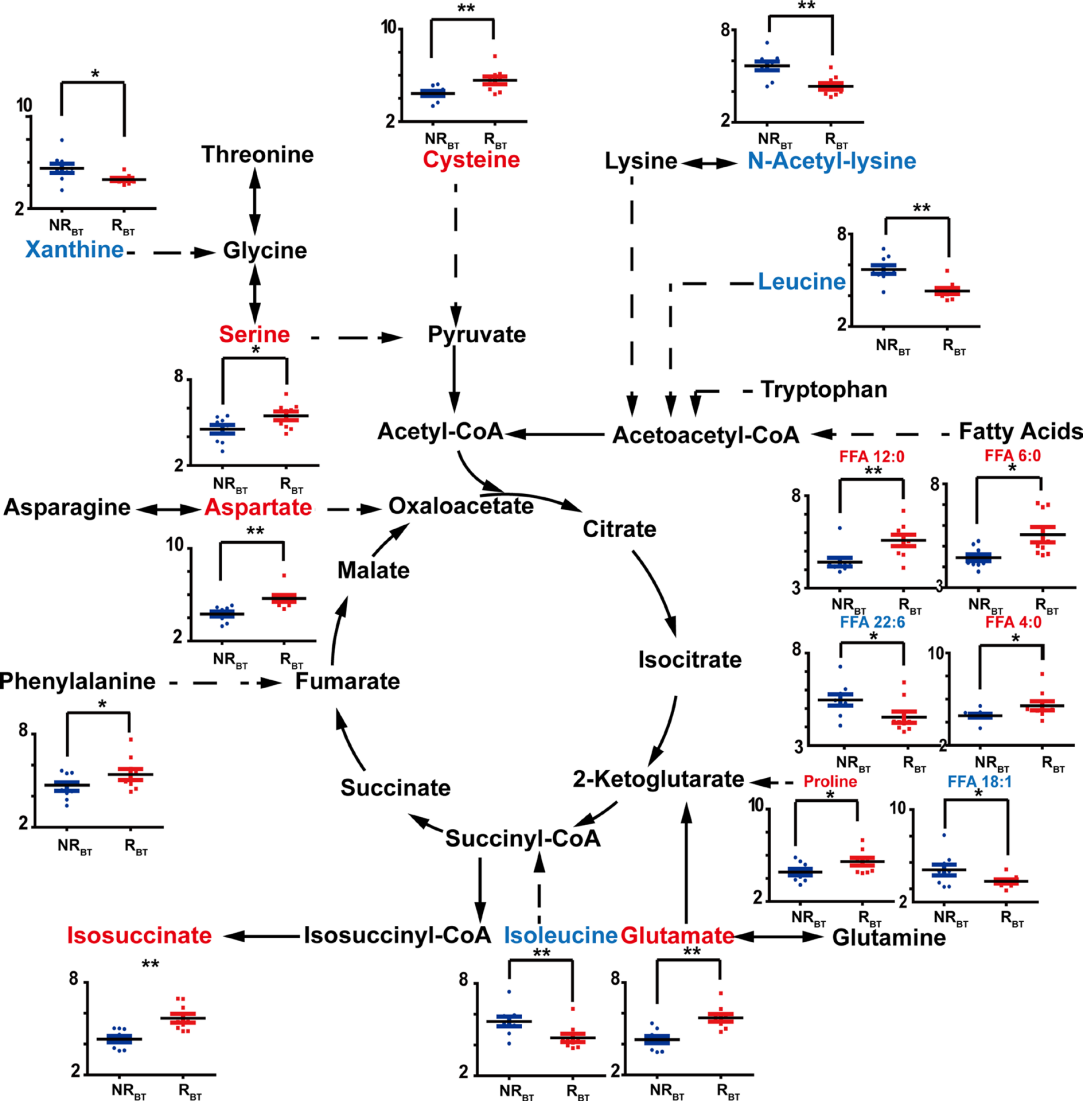
Discriminators with relative quantification (y-axis) and involved pathways for NRBT (blue bars) vs. RBT (red bars) The recurrent and non-recurrent group are separated according to the x-axis. ^*^: *p* < 0.05, ^**^: *p* < 0.01. Increase of the metabolites is represented by red color while the decrease is represented by blue color.

As shown in Figure 2E, 35 metabolites were determined to be significant in the comparison between R_BT_ and NR_BT_. Among the significant discriminators, we observed a general increase in the level of amino acids in R_BT_ samples compared with NR_BT_ samples. With regard to free fatty acids (FFA) in the serum, FFA 4:0 and FFA 12:0 were elevated in R_BT_ samples compared to NR_BT_ samples, while FFA 18:0 and FFA 22:6 (Docosahexaenoic acid, DHA) varied in the opposite direction. In addition, upregulation of two pentose phosphate pathway (PPP) metabolites, ribitol and arabitol, was observed. Also, xanthine and urate, metabolites associated with purine metabolism, were decreased in the recurrent patients. Several energy-related organic acids, including lactic acid, glycolic acid, glutaric acid, and sebacic acid, also differed between R_BT_ vs. NR_BT_. Other significant discriminators included indolelactate, benzeneacetate, and benzoate. A diagram of the relevant pathways showing the involved discriminating metabolites between R_BT_ and NR_BT_ samples is shown in Figure 3.

### Metabolomic differences after RFA therapy (AT) according to subsequent HCC recurrence

**Figure 4:**
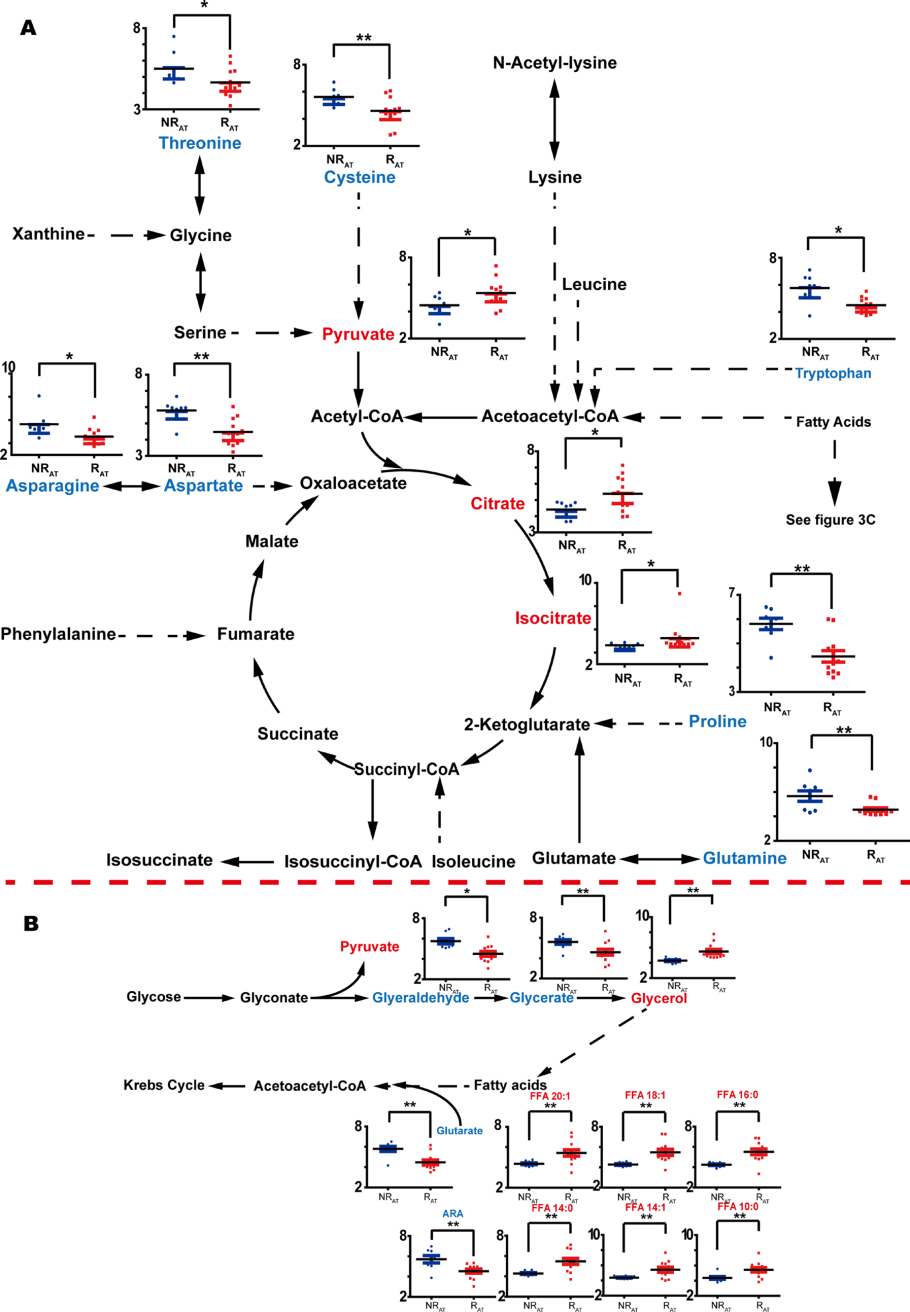
Discriminators with relative quantification (y-axis) and involved pathways for NRAT (blue bars) vs. RAT (red bars) The recurrent and non-recurrent group are separated according to the x-axis. (**A**) Discriminators without glycerolipid metabolism and fatty acids. (**B**) Discriminators with glycerolipid metabolism and the fatty acids included in the discrimination between NRAT and RAT. ^*^: *p* < 0.05, ^**^: *p* < 0.01. Increase of the metabolites is represented by red color while the decrease is represented by blue color.

For the comparison between R_AT_ and NR_AT_, 50 metabolites were observed to vary significantly (shown in Figure 2F). Among them, generally heightened fatty acids were found in the recurrent cases, except for arachidonic acid (ARA, FFA 20:4), similar to the fatty acid-related findings with BT. However, we noticed that some types of metabolites, such as amino acids, PPP-involved metabolites, and lactate, varied differently in comparison to the differences observed with the BT samples. Opposite variation directions of certain metabolites observed with BT were not observed for other metabolites such as benzoate and glutarate. Moreover, 2 metabolites involved in the TCA cycle, citrate and isocitrate, were augmented in recurrent cases after treatment but this difference was not observed in the comparison of R_BT_ vs. NR_BT_. Other significant differences related to post-treatment recurrence were found for additional metabolites including creatinine, citrulline, threonate, 2-hydroxybutyrate, 3-aminoisobuyrate, and others shown in Supplementary Table 2. Relative quantifications for some metabolite discriminators that distinguish R_AT_ from NR_AT_ are shown according to their associated pathways in Figure 4.

### Determination of potential biomarkers associated with HCC recurrence

**Figure 5:**
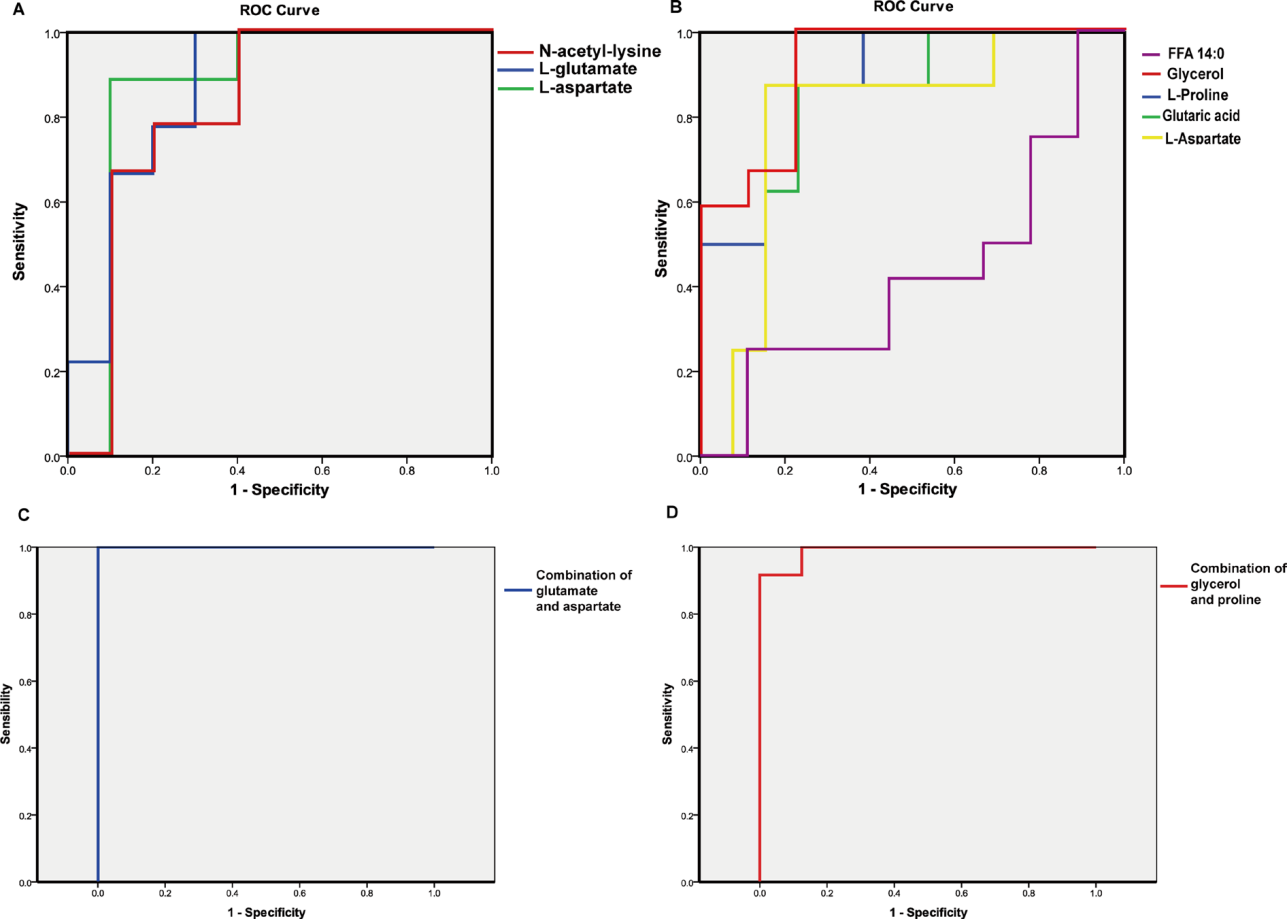
ROC curve of primary metabolite discriminators and potential combinational biomarkers of recurrence for HCV-related HCC patients (**A**-**B**) ROC for the obtained key discriminators. A: RBT vs. NRBT; B: RAT vs. NRAT. (**C**-**D**) ROC of the potential combinational biomarkers before and after RFA treatment. C: ROC of the combination of glutamate and aspartate separating RBT from NRBT; D ROC of the combination of glycerol and proline separating RAT from NRAT.

Further determination of key discriminators was achieved among the metabolites with low *P*-values. Table 2 shows the eight metabolites with *P*-values no more than 0.001 were focused on. N-acetyl-lysine, glutamate, and aspartate were characterized based on their acute variation in the R_BT_ vs. NR_BT_ comparison while aspartate, proline, glutarate, glycerol, and FFA 14:0 were characterized as the metabolites highly correlated to the prediction of HCC relapse after treatment. The receiver operating characteristic curve (ROC) for each defined metabolite was calculated to affirm its reliability for recurrence prediction (shown in Figure 5A–5B). Accordingly, apart from FFA 14:0, all the defined principal discriminators showed outstanding performance with ROCs greater than 0.80.

**Table 2:** Determination of principal metabolites that distinguished between HCV-related HCC patients with and without recurrence

	Metabolite	m/z	RT (min)	VIP	*p*-value	Fold Change	ROC	RF
**BT**	L-Glutamate	246	22.71	2.11	< 0.001	2.3	0.87	54.3%
	L-Aspartate	232	20.31	2.01	< 0.001	1.82	0.87	68.0%
	N-Acetyl-lysine	98	38.46	1.98	< 0.001	0.56	0.82	43.3%
**AT**	Glycerol	205	13.89	1.78	< 0.001	1.8	0.92	74.7%
	L-Proline	142	14.44	1.97	< 0.001	0.6	0.89	51.7%
	L-Aspartate	232	20.31	1.94	< 0.001	0.61	0.80	54.7%
	Glutaric acid	115	14.45	1.94	< 0.001	0.58	0.80	50.7%
	FFA 14:0	285	27.73	1.76	0.001	1.76	0.43	44.0%

RT: Retention Time; VIP: Variable Importance Projection; RF: Random Forest.

Validation tests of the primary discriminators were performed by the random forest (RF) method for 100 runs. For the comparison by recurrence prior to treatment, the average accuracy rate on independent tests was 80.7% ± 7.6%; the result for R_AT_ versus NR_AT_ discrimination was 82.9% ± 8.0%. Table 2 displays the probability of the presence of each discriminator in the 100 runs of the RF model. N-acetyl-lysine and FFA 14:0 were present in less than half of the RF models, which indicated that these metabolites should be rejected as candidate biomarkers. Other metabolites were proved to be more important for the two models.

Finally, after the exclusion of the two metabolites with poor performances in the RF models, the remaining metabolites were used to construct potential combinatorial biomarkers. For the model based on the samples obtained before treatment, the combination of aspartate and glutamate showed an accuracy of 100% in ROC (Figure 5C). For after treatment, glycerol and proline were the two crucial variables for prediction. Their combination showed 99% accuracy of prediction for post-operative recurrence (Figure 5D).

### Result validation with an external validation set

**Figure 6:**
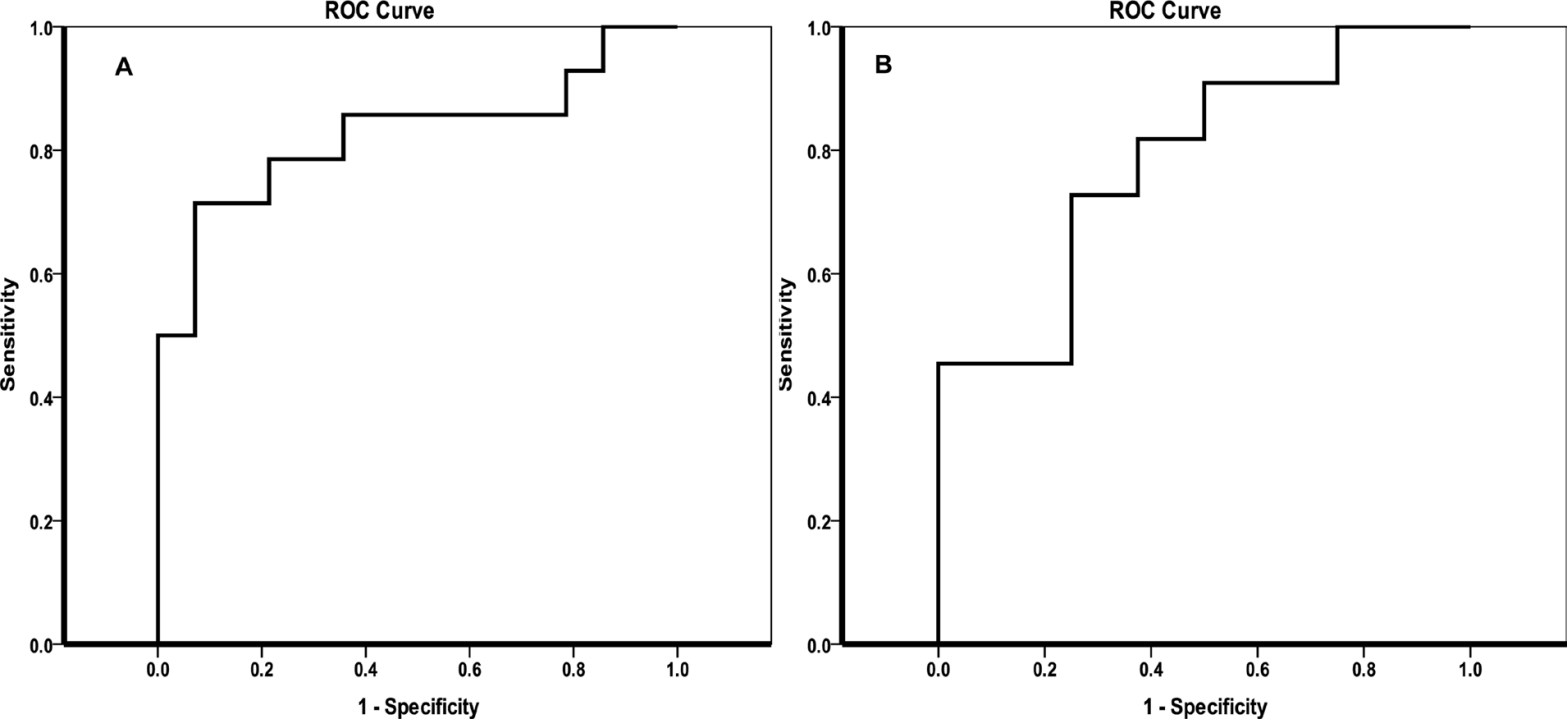
Validation of the potential biomarkers predicting the recurrence of HCC in patients with HCV by ROC curves (**A**) ROC curve for the prediction of recurrence in the BT group; (**B**) ROC curve for the prediction of recurrence in the AT group.

The ability of the two potential biomarkers to predict recurrence in samples from an external validation set was verified by another ROC analysis. As shown in Figure 6, the combination of aspartate and glutamate represents an ROC = 0.83 for the prediction of HCC recurrence in the BT samples. The ROC was equal to 0.78 for the prediction of HCC recurrence in the AT samples using the combination of glycerol and proline as predictors.

## DISCUSSION

Metabolomics is a powerful tool that can reveal subtle physiological changes in biological systems which may correspond to the development or progression of lesions and disease and prove useful for predicting the recurrence of cancer [22, 23]. In the current work, clear discriminatory factors involved in recurrence were revealed in this homogeneous cohort of HCC patients with HCV infection based on serum sample analyses before and after RFA therapy. Comprehensive metabolic alterations in patients with recurrence were detected in comparison to those without HCC relapse.

### Free fatty acids

It is notable that comprehensive turnover of free fatty acids in the recurrent patients was observed both before and after RFA treatment according to HCC recurrence status. Even-carbon chain fatty acids including long-chain and medium-chain saturated fatty acids (SFAs) and monounsaturated fatty acids (MUFAs) were increased in the NR_AT_ vs. R_AT_ comparison. Interestingly, short-chain and medium-chain fatty acids were determined to be increased in R_BT_ compared to NR_BT_. However, arachidonic acid (ARA, 20:4) and docosahexaenoic acid (DHA 22:6), which are long-chain polyunsaturated acids (PUFAs), were decreased in R_AT_ and R_BT_, respectively (Shown in Figures 3, 4A).

These data are consistent with existing data on cancer recurrence. For example, the actions of PUFAs on immune function, such as anti-inflammatory properties, are well known [24]. Therefore, depletion of PUFAs might lead to poor prognosis in this patient population due to the presence of hepatic viral infection. In addition, the HCV core protein is known to cause insulin resistance which might be an activator in the general increase in fatty acids in the blood [25]. The HCV core protein is also known to activate the liver X receptor alpha (LXRα) protein which activates inflammatory genes resulting in lipogenesis [26]. Interestingly, increased 7-hydroxycholesterol, an LXR ligand, was observed in our study in patients with recurrence (*p* < 0.05), and has also been associated with hypercholesterolemia and cancer growth and metastasis in breast cancer patients [27]. Significantly increased levels of FFAs have also been reported in a comparison between HCV-infected cells and controls [28]. Higher levels of FFAs in HCV-infected HCC patients could indicate a more activated HCV core protein. In addition, FFAs have been demonstrated to be responsible for invasion and migration of HCC cancer cells [29]. Taken together, both before and after RFA therapy, the deregulation of FFAs in the serum of HCV-infected HCC patients could at least partly account for HCC recurrence.

### Glycerolipid metabolism and energy-related metabolism

Notable upregulation of glycerol (*p <* 0.01), which is another precursor of triglyceride (TG) synthesis other than FFAs, was observed in R_AT_ patients compared with NR_AT_ patients. In addition, two upstream metabolites for glycerol, glyceraldehyde (*p* < 0.05) and glycerate (*p* < 0.01), were decreased. HCV induces insulin resistance and inhibits synthesis of TG, consistent with the accumulation of glycerol and FFAs in patients with relapse, shown in Figure 4B.

We have also observed enhanced lactate (*p* < 0.05) and pyruvate (*p* < 0.05) in recurrent patients. We have previously reported data supporting upregulation of lactate as a potential biomarker for HCC recurrence [30]. This is due to the high energy demand associated with HCC cell emergence [31]. In addition, it is well known that the HCV core protein activates hypoxia-inducible factor 1 (HIF1), which promotes the formation of a hypoxic environment for the development of HCC [3]. Consistent with these data, we observed increases in two metabolites in the TCA cycle, citrate (*p* < 0.05) and isocitrate (*p* < 0.05) which were only found in patients after RFA who subsequently experienced recurrence. This may be attributable to increased energy demand associated with tumor growth.

### Amino acids metabolism

According to our analyses before and after RFA therapy, amino acid levels were of great importance for distinguishing between patients who had or had not presented with recurrence (shown in Figures 3 and 4A). In line with our data for the discriminatory analyses of recurrence in patients after treatment, the downregulation of various amino acids presented in Figure 4 had been previously reported to indicate a less optimistic prognosis in HCC patients [12, 16, 18, 32–35]. This was primarily attributed to the comprehensive protein depletion associated with clinical treatment and the upregulation of anabolism prior to relapse. However, in contrast to the results in the AT evaluation, except for two branched chain amino acids (BCAA), a reverse trend was observed in a number of amino acids in the NR_BT_ vs R_BT_ analysis. Previous reports have shown that increased serine and threonine pathway are considered to be an important sign of poor prognosis in cancer [16, 18]. Upregulation of proline and cysteine pathways have also been associated with proliferation and cell cycle in HCC [36]. Elevated levels of glutamate and aspartate might be related to the deregulation of aminotransferases such as γ-glutamyl transpeptidase (GGT) and aspartate aminotransferase (AST) in the case of HCC [37, 38].

### Pentose phosphate metabolism

Two PPP-involved metabolites, arabitol and ribitol, were found significantly increased in the comparison of R_BT_ to NR_BT_. Being related to the synthesis of nucleosides, upregulation of the pentose pathway might be due to dynamic proliferation of DNA before RFA therapy in patients who subsequently developed HCC recurrence. On the contrary, after the tumor was removed, significant declines, not only in the above two metabolites but also in ribonic acid and xylonic acid, were observed in R_AT_ compared to NR_AT_. Given that TCA cycle-associated metabolites and lactate were found elevated in the recurrent group after RFA therapy, this decrease in PPP metabolites might result from down-regulation of the energy supply from this pathway.

### Other significantly varied metabolites in recurrent patients

Other metabolites, listed in Supplementary Table 2, were also significantly altered in R_BT_ patients compared with those in the NR_BT_ group. Declines in xanthine and urate may be attributable to the consumption of purines during the replication of DNA. Elevated methylmalonate is likely associated with the upregulation of the TCA cycle and the degradation of BCAAs. The acetylation of lysine has been reported to be attenuated in another cancer [39], in accordance with our results.

Metabolites such as citrulline and oxalate were also able to distinguish between NR_AT_ and R_AT_. Citrulline is synthesized in the liver, and decreased citrulline indicates altered liver function and decreased citrulline synthesis from argininosuccinate [40]. Oxalate has also been reported to be augmented in recurrent patients when compared with non-recurrent patients [30].

### Determination of combinatorial biomarker of recurrence for HCV-related HCC patients

The primary discriminators related to recurrence were defined, as shown in Figure 5. In particular, the combination of aspartate and glutamate shows the potential to predict recurrence prior to treatment. As discussed above, previous studies have reported the predictability of HCC recurrence by AST and GGT. This data provides evidence that the two amino acids which are modulated by these two aminotransferases may be also significant indicators for recurrence before clinical treatment. Cao et al. recently demonstrated differential tissue metabolomic data for alcoholic liver disease-induced HCC compared to hepatitis virus-induced HCC and showed that virus-induced HCC was correlated with significant variations in aspartate and glutamate [41]. The results also indicated that these variations might be specific to the development of HCV-related HCC.

After small HCC tumors were removed by RFA treatment, we noted a remarkable upregulation of glycerol accompanied by increased levels of various fatty acids that should contribute to HCV-induced insulin resistance and hinder the synthesis of TG. The combination of glycerol and proline was shown to be a powerful predictor of HCC recurrence after treatment. This deregulation of glycerol involved pathways was also in accordance with what was reported by Cao et al. [41]. The validation with external samples showed the predictability of the defined potential biomarkers were acceptable both before and after RFA therapy.

### Limitation

This study has some limitations. First, the number of studied patients was restricted by the conditions of the screening criteria. These results should be validated in a larger cohort of HCV-HCC patients. Second, even though all the enrolled patients were diagnosed with small HCC, there were still differences in tumor size among the patients, which might be a factor influencing the metabolic profile. Also, as metabolomic analyses targeting lesions of HCC might be more specific than serum profiling investigations, a subsequent study will be performed with a larger cohort based on tumor tissue metabolomics.

## MATERIALS AND METHODS

### Ethics statement

The Institutional Review Board of Jean Verdier University Hospital approved the protocol and the French Research Delegation Office approved the creation of a dedicated bio-collection for the patients who have been included in the protocol. The CNIL (National Informatics and Liberty Commission) also approved the creation of both biocollection and database. Written informed consent was obtained directly from each patient at inclusion. All methods were performed in accordance with the relevant guidelines and regulations.

### Sample collection

All serum samples were collected by Jean Verdier Hospital, Bondy, France (F93140) and were stored by the “CRB (liver disease biobank) Groupe Hospitalier Paris Seine-Saint-Denis BB-0033–00027”. They were derived from the same cohort of HCC patients included in our previous metabolomic explorations [21]. From January 2002 to December 2012, all cirrhotic patients referred to our institution for the management of HCC by percutaneous ablation were considered. Among them, 349 underwent monopolar or multipolar RFA and had available frozen sera samples before and after the procedure. The following inclusion criteria were considered: 1) biopsy-proven cirrhosis, 2) tumor stage classified as Barcelona Clinic Liver Cancer (BCLC) stage 0 [1], 3) availability of frozen serum samples before and after RFA, 4) acceptance of regular follow-up. Only HCV-related HCC patients with active viral replication were selected based on these criteria and were included in the present analyses.

HCC was diagnosed according to the Barcelona criteria: histological evidence or convergent demonstration of a focal lesion more than 2 cm in size and with arterial hyper-vascularization by two different imaging techniques, or the combination of one imaging technique showing this morphological aspect with an AFP level superior to 400 ng/mL. The decision to treat patients with RFA was made by a multidisciplinary team according to international recommendations [42]. All RFAs were percutaneously performed by the same operator under ultrasound guidance and general anaesthesia.

Clinical, biological, and radiological data of patients were recorded on the day of the first blood collection which was considered as the inclusion date. Response was assessed at one month after RFA, then every three months for two years, then every six months, by three-phase contrast CT or MR examination, and AFP serum level measurement. A tumor was considered as completely ablated if no nodular or irregular enhancement adjacent to the ablation zone was visible at the arterial phase. Local or distant tumor recurrences were assessed. A total of 46 patients were enrolled in the present study, among whom 24 experienced recurrence during the first two years after the RFA procedure. Twenty-one enrolled patients were included in the training set and 25 patients comprised the validation test. Along with follow-up, the first sampling took place within 14 days before RFA therapy, noted as samples obtained before operation, while the samples obtained after treatment were drawn two months after the RFA procedure.

### Chemicals

The applied chemicals have been listed in the Supplementary Information (2).

### Sample preparation

The samples in the training set and the validation set were analyzed in a stepwise manner. All of them were initially stored at -80°C until sample preparation, which was performed on ice. An equal aliquot was extracted from each serum sample in order to make up a pool of samples, from which 16 QC samples in the training set and 9 QC samples in the validation set were subsequently taken. For the real samples, a volume of 100 μL was extracted from each sample into an Eppendorf tube to which 400 μL of methanol solution (methanol: H_2_O = 4:1) was added to precipitate proteins. The liquid supernatant (440 μL) from each tube was withdrawn after centrifugation at 15 000 x g for 15 minutes at 4°C. Extracted samples were then freeze-dried.

During the redissolution process, 100 μL of methoxyamine solution (20 mg/mL in pyridine) was added to each sample. The samples were incubated in a water bath at 37°C for 1.5 hour to allow the derivatization reaction to occur. This was followed by addition of 80 μL of MSTFA and additional water bath incubation for one hour. After centrifuged, the supernatant was taken for GC-MS analysis. The samples in the validation set were similarly prepared except that 10 μL of L-norvaline was added as an internal standard.

### GC-MS analysis

A Shimadzu GC-MS 2010 was used for metabolic profiling of each sample. A capillary column (30 m *250 μm *0.25 μm) and an electron ionization (EI) source were employed. The carrier gas was helium with a flow rate of 1.2 mL/min through the chromatography column. The split ratio was set to 10:1. For the training set, the gradient temperature program was set as follows: 70°C at the beginning (for 3 minutes), then gradually increased to 220°C by an increase of 4°C/min, and then heated up to 300°C with a gradient of 8°C/min, held for 10 minutes. The voltage of the detector was set at 1.1 kV. The temperature of the injection port and the transfer line was kept at 280°C. The temperature of the ion source was 230°C. The scan field was set between 33 and 600 m/z.

For the validation set, samples were analyzed in the same apparatus as that used for the training set samples. The metabolite discriminators defined by the training set, as well as the internal standard L-norvaline, were analyzed in selective ion monitoring (SIM) mode. The targeted characteristic ions defined by the training set were specifically evaluated in the experiment. The retention time ranges corresponding to the above ions are displayed in Supplementary Table 3, while the exact recorded ionic retention times are shown in Supplementary Table 4.

The gradient temperature program for the validation set also differs from that for the training set: This was started at 70°C for the first 3 minutes, and the temperature was increased to 180°C by 5°C/min, then went up to 310°C by 20°C/min, and was retained for 5 minutes. The even time was set to 0.2 s.

Several blank samples (without a sample in the vial) were tested before the sample analysis to remove residues remaining in the column. Before the analysis of the real samples, several QC samples were analyzed to confirm the stability of the instrument. Each injection was a 1 μL of droplet from each sample. One QC sample was inserted after every 6 non-control samples in the sequence of analysis to monitor the reproducibility and stability of the method. Once the spectra of all the samples were recorded, a light diesel sample was subsequently analyzed for acquiring the retention index (RI).

### Data processing

Pretreatments such as deconvolution, peak matching, and retention time alignment of the spectra were performed with the help of Leco ChromaTOF software (St. Joseph MI, version 3.25) and XCMS (xcmsonline.scripps.edu). The assignment of the ion peaks was achieved by matching to a library (NIST, Mainlib, Replib, Wiley and Feihn). The assignment of peaks was based on m/z and the calculated RI which was associated with the retention time. The qualification for most of the principal discriminators was confirmed by analyzing the correspondent reference substance. For cases in which several peaks corresponded to a single metabolite, we retained the one with the lowest RSD.

The recording of the area of each identified ion peak was regarded as the relative quantification for its corresponding metabolite. A data set recording the area for the ion peaks was generated. Peaks were screened by the same method performed in one of our previous studies [43].

PCA and PLS-DA were done using SIMCA-P software (version 11, Umetrics, Umeå, Sweden). Heat maps were displayed by the MultiExperiment Viewer (Mev, version 4.9.0, Dana-Farber Cancer Institute, MA, USA). Other statistical analyses were performed using SPSS (version 19, IBM, Chicago, USA). The acquisition of spectra for some available reference substances served to confirm the qualification of most of the discriminators.

To investigate the predictive ability of the defined candidate biomarkers of HCC recurrence, the validation of defined principal metabolites was performed by feature selection of RF. The calculation was performed according to an algorithm written in the C++ language. For the discrimination between recurrent and non-recurrent HCV-HCC patients, two-thirds of the samples were used as training data while the remaining one-third of the samples acted as the validation set. Both of the RF analyses were run 100 times, the frequency of the presence of a certain discriminator in all runs was obtained. Discriminators with a frequency of presence superior to 50% were considered to be potential biomarkers.

Binary logistic regression (BLR) with the “condition: forward” method was used to select the potential biomarkers among the discriminators. The data from the obtained discriminators were then resubmitted to for BLR with the “enter” method. The probability representing the combination of the discriminators was calculated for each sample and the probability was tested in the ROC.

## CONCLUSIONS

In the present study, we have investigated dynamic metabolic serum changes, before and after RFA for HCC, taking into account the presence of subsequent recurrence. In HCC patients with HCV infection, clear separation by recurrence was found both before and after RFA treatment. Further analysis showed a general increase of various fatty acids in the patients experiencing recurrence as compared with patients who did not have recurrence, which may be associated with HCV infection-induced deregulation. Inconsistent alterations in amino acid and PPP pathways were revealed between pre-treatment and post-treatment patients. We suggest that specific metabolic variations observed prior to the suppression of the tumor in patients who subsequently experienced recurrence were linked to DNA replication and tumor proliferation. However, observed declines in amino acid levels and deregulation of energy-related metabolites, especially TCA cycle-involved metabolites, may be associated with protein depletion after clinical treatment and increased anabolism for tumorigenesis. External validation data suggest that the combination of glutamate and aspartate and the combination of glycerol and proline may be potential biomarkers for the prediction of HCC recurrence before and after RFA treatment, respectively.

## SUPPLEMENTARY MATERIALS FIGURES AND TABLES





## Abbreviations

HCC: Hepatocellular carcinoma
HCV: Hepatitis C-Virus
RFA: Radiofrequency Ablation
GC-MS: Gas Chromatography-Mass Spectrometry
TCA: Tricarboxylic Acid
RF: Random Forest
AFP: Alpha Fetoprotein
LC-MS: Liquid Chromatography-Mass Spectrometry
QC: Quality Control
RSD: Relative Standard Deviation
ROC: Receiver Operating Characteristic Curve
BT: Before Treatment
AT: After Treatment
R: Recurrent patients
NR: Non-Recurrent patients
PCA: Principal Component Analysis
PLS-DA: Partial Least Square-Discriminant Analysis
VIP: Variable Importance in the Projection
DHA: Docosahexaenoic Acid
PPP: Pentose Phosphate Pathway
ARA: Arachidonic Acid
FFA: Free Fatty Acids
SFAs: Saturated Fatty Acids
MUFAs: Monounsaturated Fatty Acids
PUFAs: Polyunsaturated Acids
LXRα: Liver X Receptor alpha
HIF1: Hypoxia-Inducible Factor 1
GGT: γ-Glutamyl Transpeptidase
AST: Aspartate aminotransferase
TG: Triglyceride
BCLC: Barcelona Clinic Liver Cancer
MSTFA: N-methyl-N-, trimethylsilyl)trifluoroacetamide
SIM: Selective Ion Monitoring
RI: Retention Index
BLR: Binary Logistic Regression

## References

[1] Llovet JM, Burroughs A, Bruix J (2003). Hepatocellular carcinoma. The Lancet.

[2] Bruix J, Gores GJ, Mazzaferro V (2014). Hepatocellular carcinoma: clinical frontiers and perspectives. Gut.

[3] Arzumanyan A, Reis HM, Feitelson MA (2013). Pathogenic mechanisms in HBV- and HCV-associated hepatocellular carcinoma. Nat Rev Cancer.

[4] Livraghi T, Goldberg SN, Lazzaroni S, Meloni F, Solbiati L, Gazelle GS (1999). Small hepatocellular carcinoma: Treatment with radio-frequency ablation versus ethanol injection. Radiology.

[5] El-Serag HB (2012). Epidemiology of viral hepatitis and hepatocellular carcinoma. Gastroenterology.

[6] Levrero M (2006). Viral hepatitis and liver cancer: the case of hepatitis C. Oncogene.

[7] Davila JA, Morgan RO, Shaib Y, McGlynn KA, El–Serag HB (2004). Hepatitis C infection and the increasing incidence of hepatocellular carcinoma: a population-based study. Gastroenterology.

[8] Llovet JM, Bruix J (2008). Novel advancements in the management of hepatocellular carcinoma in 2008. J Hepatol.

[9] Mamas M, Dunn WB, Neyses L, Goodacre R (2011). The role of metabolites and metabolomics in clinically applicable biomarkers of disease. Arch Toxicol.

[10] Xia J, Broadhurst DI, Wilson M, Wishart DS (2013). Translational biomarker discovery in clinical metabolomics: an introductory tutorial. Metabolomics.

[11] Zhou L, Ding L, Yin P, Lu X, Wang X, Niu J, Gao P, Xu G (2012). Serum metabolic profiling study of hepatocellular carcinoma infected with hepatitis B or hepatitis C virus by using liquid chromatography-mass spectrometry. J Proteome Res.

[12] Zeng J, Yin P, Tan Y, Dong L, Hu C, Huang Q, Lu X, Wang H, Xu G (2014). Metabolomics study of hepatocellular carcinoma: discovery and validation of serum potential biomarkers by using capillary electrophoresis-mass spectrometry. J Proteome Res.

[13] Nahon P, Amathieu R, Triba MN, Bouchemal N, Nault JC, Ziol M, Seror O, Dhonneur G, Trinchet JC, Beaugrand M, Le Moyec L (2012). Identification of serum proton NMR metabolomic fingerprints associated with hepatocellular carcinoma in patients with alcoholic cirrhosis. Clin Cancer Res.

[14] Zhou L, Liao Y, Yin P, Zeng Z, Li J, Lu X, Zheng L, Xu G (2014). Metabolic profiling study of early and late recurrence of hepatocellular carcinoma based on liquid chromatography-mass spectrometry. J Chromatogr B Analyt Technol Biomed Life Sci.

[15] Nezami Ranjbar MR, Luo Y, Di Poto C, Varghese RS, Ferrarini A, Zhang C, Sarhan NI, Soliman H, Tadesse MG, Ziada DH, Roy R, Ressom HW (2015). GC-MS Based Plasma Metabolomics for Identification of Candidate Biomarkers for Hepatocellular Carcinoma in Egyptian Cohort. PLoS One.

[16] Fitian AI, Nelson DR, Liu C, Xu Y, Ararat M, Cabrera R (2014). Integrated metabolomic profiling of hepatocellular carcinoma in hepatitis C cirrhosis through GC/MS and UPLC/MS-MS. Liver Int.

[17] Budhu A, Roessler S, Zhao X, Yu Z, Forgues M, Ji J, Karoly E, Qin LX, Ye QH, Jia HL, Fan J, Sun HC, Tang ZY (2013). Integrated metabolite and gene expression profiles identify lipid biomarkers associated with progression of hepatocellular carcinoma and patient outcomes. Gastroenterology.

[18] Chen T, Xie G, Wang X, Fan J, Qiu Y, Zheng X, Qi X, Cao Y, Su M, Wang X, Xu LX, Yen Y, Liu P (2011). Serum and Urine Metabolite Profiling Reveals Potential Biomarkers of Human Hepatocellular Carcinoma. Molecular & Cellular Proteomics.

[19] Patterson AD, Maurhofer O, Beyoglu D, Lanz C, Krausz KW, Pabst T, Gonzalez FJ, Dufour JF, Idle JR (2011). Aberrant lipid metabolism in hepatocellular carcinoma revealed by plasma metabolomics and lipid profiling. Cancer Res.

[20] Wu H, Xue R, Dong L, Liu T, Deng C, Zeng H, Shen X (2009). Metabolomic profiling of human urine in hepatocellular carcinoma patients using gas chromatography/mass spectrometry. Anal Chim Acta.

[21] Goossens C, Nahon P, Le Moyec L, Triba MN, Bouchemal N, Amathieu R, Ganne-Carrie N, Ziol M, Trinchet JC, Sellier N, Diallo A, Seror O, Savarin P (2016). Sequential Serum Metabolomic Profiling after Radiofrequency Ablation of Hepatocellular Carcinoma Reveals Different Response Patterns According to Etiology. J Proteome Res.

[22] Asiago VM, Alvarado LZ, Shanaiah N, Gowda GA, Owusu-Sarfo K, Ballas RA, Raftery D (2010). Early Detection of Recurrent Breast Cancer Using Metabolite Profiling. Cancer Research.

[23] Cao MD, Sitter B, Bathen TF, Bofin A, Lonning PE, Lundgren S, Gribbestad IS (2012). Predicting long-term survival and treatment response in breast cancer patients receiving neoadjuvant chemotherapy by MR metabolic profiling. NMR Biomed.

[24] Calder PC (2010). Omega-3 fatty acids and inflammatory processes. Nutrients.

[25] Li HC, Ma HC, Yang CH, Lo SY (2014). Production and pathogenicity of hepatitis C virus core gene products. World J Gastroenterol.

[26] Lima-Cabello E, Garcia-Mediavilla MV, Miquilena-Colina ME, Vargas-Castrillon J, Lozano-Rodriguez T, Fernandez-Bermejo M, Olcoz JL, Gonzalez-Gallego J, Garcia-Monzon C, Sanchez-Campos S (2011). Enhanced expression of pro-inflammatory mediators and liver X-receptor-regulated lipogenic genes in non-alcoholic fatty liver disease and hepatitis C.. Clin Sci (Lond).

[27] Nelson ER, Wardell SE, Jasper JS, Park S, Suchindran S, Howe MK, Carver NJ, Pillai RV, Sullivan PM, Sondhi V, Umetani M, Geradts J, McDonnell DP (2013). 27-Hydroxycholesterol Links Hypercholesterolemia and Breast Cancer Pathophysiology. Science.

[28] Roe B, Kensicki E, Mohney R, Hall WW (2011). Metabolomic Profile of Hepatitis C Virus-Infected Hepatocytes. PLoS ONE.

[29] Nath A, Li I, Roberts LR, Chan C (2015). Elevated free fatty acid uptake via CD36 promotes epithelial-mesenchymal transition in hepatocellular carcinoma. Sci Rep.

[30] Ye G, Zhu B, Yao Z, Yin P, Lu X, Kong H, Fan F, Jiao B, Xu G (2012). Analysis of urinary metabolic signatures of early hepatocellular carcinoma recurrence after surgical removal using gas chromatography-mass spectrometry. J Proteome Res.

[31] Vander Heiden MG, Cantley LC, Thompson CB (2009). Understanding the Warburg effect: the metabolic requirements of cell proliferation. Science.

[32] DeBerardinis RJ, Cheng T (2010). Q’s next: the diverse functions of glutamine in metabolism, cell biology and cancer. Oncogene.

[33] Long J, Wang H, Lang Z, Wang T, Long M, Wang B (2011). Expression level of glutamine synthetase is increased in hepatocellular carcinoma and liver tissue with cirrhosis and chronic hepatitis B.. Hepatol Int.

[34] Zhang B, Dong LW, Tan YX, Zhang J, Pan YF, Yang C, Li MH, Ding ZW, Liu LJ, Jiang TY, Yang JH, Wang HY (2013). Asparagine synthetase is an independent predictor of surgical survival and a potential therapeutic target in hepatocellular carcinoma. Br J Cancer.

[35] Loayza-Puch F, Rooijers K, Buil LC, Zijlstra J, Oude Vrielink JF, Lopes R, Ugalde AP, van Breugel P, Hofland I, Wesseling J, van Tellingen O, Bex A, Agami R (2016). Tumour-specific proline vulnerability uncovered by differential ribosome codon reading. Nature.

[36] De Giorgi V, Buonaguro L, Worschech A, Tornesello ML, Izzo F, Marincola FM, Wang E, Buonaguro FM (2013). Molecular Signatures Associated with HCV-Induced Hepatocellular Carcinoma and Liver Metastasis. PLoS ONE.

[37] Song P, Inagaki Y, Wang Z, Hasegawa K, Sakamoto Y, Arita J, Tang W, Kokudo N (2015). High Levels of Gamma-Glutamyl Transferase and Indocyanine Green Retention Rate at 15 min as Preoperative Predictors of Tumor Recurrence in Patients With Hepatocellular Carcinoma. Medicine (Baltimore).

[38] Poon RT, Fan ST, Ng IO, Lo CM, Liu CL, Wong J (2000). Different risk factors and prognosis for early and late intrahepatic recurrence after resection of hepatocellular carcinoma. Cancer.

[39] Zhao D, Zou SW, Liu Y, Zhou X, Mo Y, Wang P, Xu YH, Dong B, Xiong Y, Lei QY, Guan KL (2013). Lysine-5 acetylation negatively regulates lactate dehydrogenase A and is decreased in pancreatic cancer. Cancer Cell.

[40] Wheatley DN, Kilfeather R, Stitt A, Campbell E (2005). Integrity and stability of the citrulline-arginine pathway in normal and tumour cell lines. Cancer Lett.

[41] Cao D, Cai C, Ye M, Gong J, Wang M, Li J, Gong J (2017). Differential metabonomic profiles of primary hepatocellular carcinoma tumors from alcoholic liver disease, HBV-infected, and HCV-infected cirrhotic patients. Oncotarget.

[42] Bruix J, Sherman M (2011). Management of hepatocellular carcinoma: an update. Hepatology.

[43] Liu Z, Yin P, Amathieu R, Savarin P, Xu G (2016). Application of LC-MS-based metabolomics method in differentiating septic sur*vivo*rs from non-sur*vivo*rs. Anal Bioanal Chem.

