# Supplementary material for: Determination of candidate metabolite biomarkers associated with recurrence of HCV-related hepatocellular carcinoma
Source: Oncotarget. 2017 Dec 15;9(5):6245–58. doi: 10.18632/oncotarget.23500 (PMC5814209; doi:10.18632/oncotarget.23500)
Supplement: Supplementary file 1 [file oncotarget-09-6245-s001.pdf]

Determination of candidate metabolite biomarkers associated with recurrence of HCV-related hepatocellular carcinoma

SUPPLEMENTARY MATERIALS

Peak screening by RSD

After the deconvolution and peak alignment for the QC samples, 459 peaks were initially detected. After removing the peaks with more than 20% null area, it was shown that 87% of the peaks were found stable with their RSD of area inferior to 30%, occupying 94% of total peak area. Other peaks with RSD superior to 30% were therefore excluded.

The derivatization reagents, pyridine, N-methyl-N-(trimethylsilyl)-trifluoroacetamide (MSTFA) and the internal standard L-Norvaline were purchased from Sigma-Aldrich China Inc. (Shanghai, China). The dichloromethane was bought from Merck (Darmstadt, Germany). The ultrapure water was obtained by Milli-Q water purification system (Millipore, USA). Other chemicals used for reference substance were listed in the supplementary information.

2-Hydroxybutyrate, 3-Hydroxybutyrate, 3-aminoisobutyrate, 7-hydroxycholesterol, benzoate, citrate, isocitrate, gluconate, glyceraldehyde, glycerate, glycerol, glycolate, hydroxyisovalerate, hydroxylamine, isosuccinate, lactate, threonate, oxalate, urate, xanthine, citrulline, L- asparagine, L-aspartate, L-cysteine, L-glutamine, L-glutamate, L-isoleucine, L-leucine, L-proline, L-threonine, L-serine, L-tryptophan and N-acetyl-L-lysine were purchased from Sigma-Aldrich China Inc. (Shanghai, China); D-arabitol, D-ribitol, butanoic acid (FFA 4:0), hexanoic acid (FFA 6:0), decanoic acid (FFA 10:0), dodecanoic acid (FFA 12:0), pentadecanoic acid (FFA 15:0), palmitic acid (FFA 16:0), octadecanoic acid (FFA 18:0), 11-Eicosenoic acid (FFA 20:1), Arachidonic acid (FFA 20:4) and Docosahexaenoic acid (FFA 22:6) were offered by the J&K Scientific Ltd. (Beijing, China).

Supplementary Table 1: Relative standard deviation (RSD) for all the detected characteristic ions

Metabolites	m/z	Retention time (min)	RSD
L-Norvaline 1	72	9.34	0.255
L-Proline 1	70	11	0.359
L-Norvaline 2	144	13.05	0.000
Glycerol	205	13.93	0.230
L-Proline 2	142	14.48	0.196
Aspartate 1	160	17.86	0.139
Aspartate 2	232	20.33	0.361
Glutamate 1	84	20.61	0.290
Glutamate 2	246	22.72	0.761

For the peak normalization by the internal standard, 2 characteristicions for L-norvaline (m/z = 72, m/z = 144) were detected. The ion m/z = 144 was ultimately chosen as the reference of peak normalization because it brought a RSD = 0.17, which was calculated by the peak area in all the 9 QC samples, lower than that for m/z = 72 (RSD = 0.25). After all the peak areas were normalized, RSD for the targeted ions in each QC sample were obtained as shown. For one metabolite with two detected characteristicions, the ion with a lower RSD than the other (presented in bold) was chosen for the following analyses.

Supplementary Table 2: Significant altered metabolites in discrimination between recurrent HCV-HCC patients and non-recurrent HCV-HCC patients. See_ Supplemental_Table 2

Supplementary Table 3: Targeted scan period and ions for the validation experience.

Scan time (min)	Targeted Ions (m/z)
9～12	72, 70
12～16	144, 205, 142
17～19	160
19～21	232, 84
21～24	142, 246

The listed ions are the ions characterize the metabolites norvaline (targeted ion: 72/144), glutamate (84/246), aspartate (160,232), proline (70,142) and glycerol (205).

Supplementary Table 4: Definition of the characteristicions for the targeted metabolites

Metabolites	Retention time (min)	m/z
Aspartate	18.16	160
	20.63	232
	11.3	70
Proline	14.75	142
	20.04	142
Glutamate	20.91	84
	23.02	246
Glycerol	14.21	205
L-Norvaline	9.64	72
	13.35	144

The information was obtained by analyzing the corresponding standards.

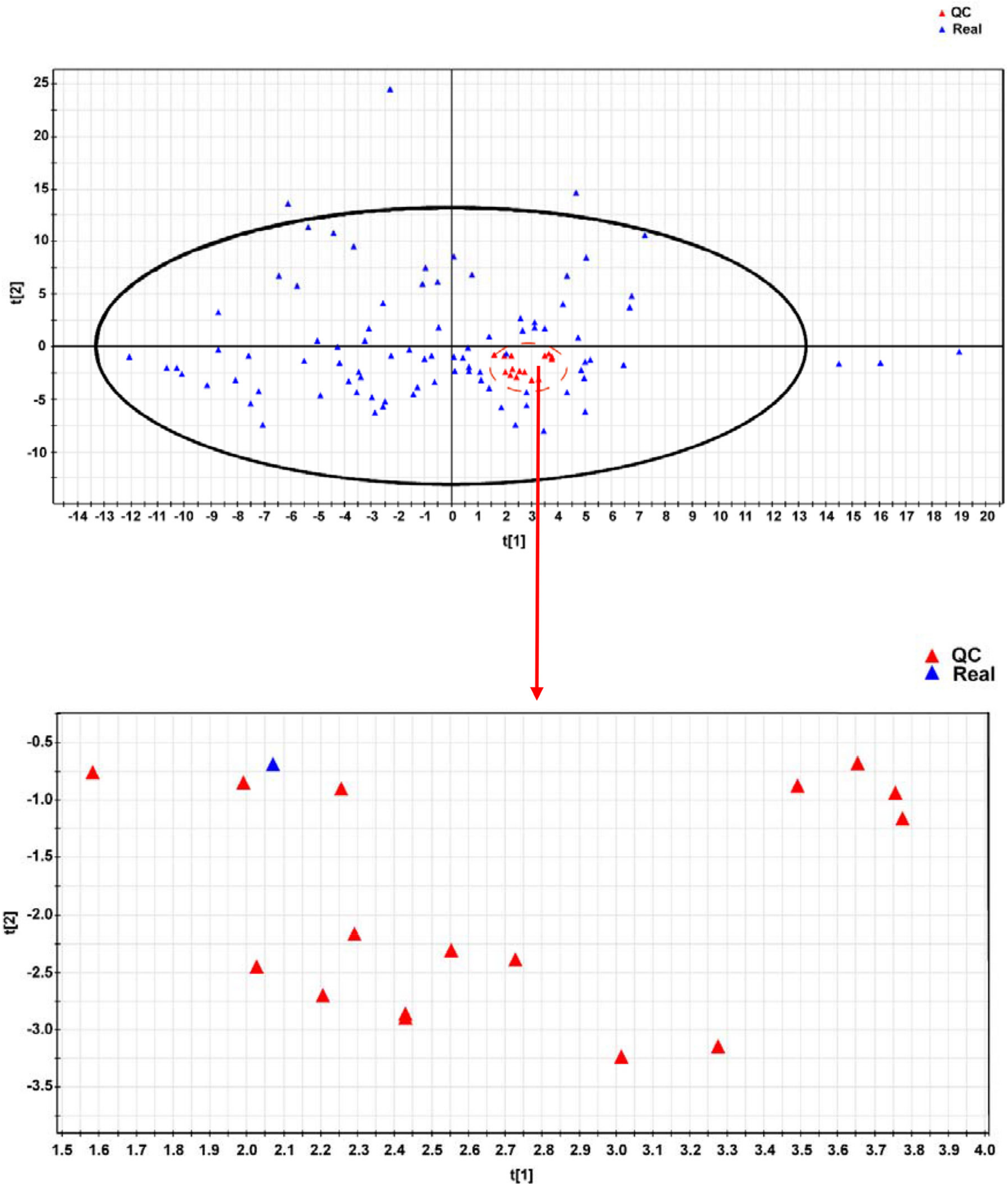

Supplementary Figure 1: Score plot of PCA presenting the comparison between the QC samples (red and circled triangles) and the real samples (blue triangles).

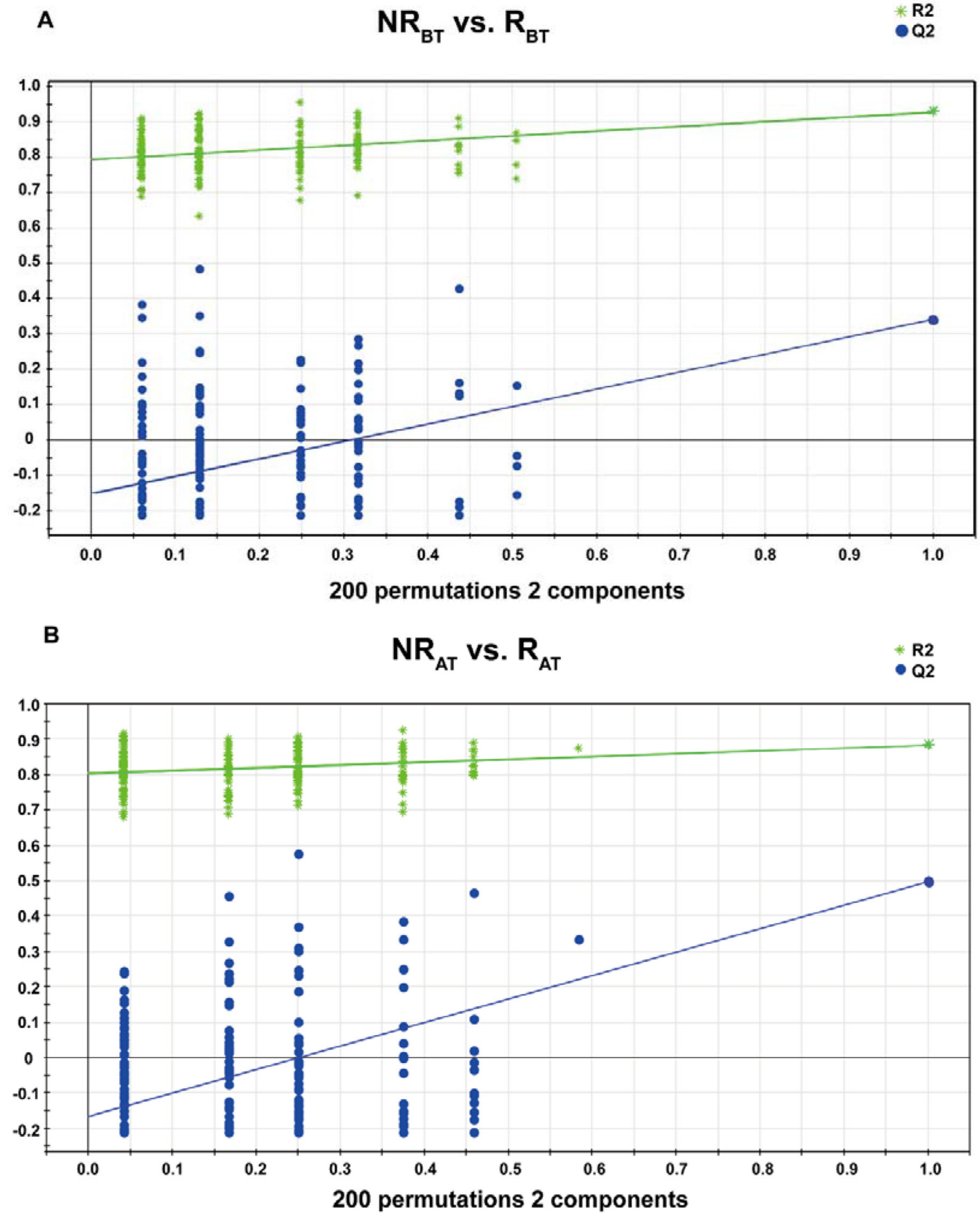

Supplementary Figure 2: Cross-validation for the two PLS-DA models separating the recurrent and the non-recurrent HCC patients with HCV. (**A**) Model validation for NRBT vs RBT (**B**) Model validation for NRAT vs RAT. The samples were permutated into a different order from that in the established model for 200 times. The green triangles stand for the obtained R^2 value and the blue squares stand for the obtained Q^2 value by the 200 permutations. The Y-axis represents R^2 and Q^2 value for every model while the X-axis represents the correlation coefficient between original and permuted response data.
