# Supplementary material for: Determination of candidate metabolite biomarkers associated with recurrence of HCV-related hepatocellular carcinoma
Source: Oncotarget. 2017 Dec 15;9(5):6245–58. doi: 10.18632/oncotarget.23500 (PMC5814209; doi:10.18632/oncotarget.23500)
Supplement: Supplementary file 2 [file oncotarget-09-6245-s002.docx]

Supplementary Table-2. Significant altered metabolites in discrimination between recurrent HCV-HCC patients and non-recurrent HCV-HCC patients. BT: before RFA therapy; AT: after RFA therapy. TR: time of retention. FC: fold change, calculated by the ratio of R/NR. *: metabolites confirmed by the standard reference substance.

| Subgroup | TR | QN | Metabolites | P-value | FC |
| --- | --- | --- | --- | --- | --- |
| BT | 7.95 | 117 | Lactate* | 0.021 | 0.81 |
|  | 8.32 | 173 | FFA 6:0* | 0.009 | 1.76 |
|  | 8.39 | 147 | Glycolic acid* | 0.027 | 1.3 |
|  | 9.74 | 131 | 2-Hydroxybutyrate* | 0.006 | 0.55 |
|  | 10.60 | 86 | L-Leucine* | 0.001 | 0.59 |
|  | 11.17 | 86 | L-isoleucine* | 0.009 | 0.63 |
|  | 13.39 | 132 | L-Serine* | 0.021 | 1.37 |
|  | 14.53 | 164 | Benzeneacetate | 0.010 | 1.65 |
|  | 15.01 | 147 | Isosuccinate* | 0.001 | 1.47 |
|  | 17.47 | 147 | Unknown 3 | 0.006 | 1.98 |
|  | 17.51 | 373 | Sebacic acid | 0.030 | 1.31 |
|  | 17.59 | 103 | FFA 4:0 | 0.038 | 1.44 |
|  | 20.31 | 232 | L-Aspartate* | 0.001 | 1.82 |
|  | 20.41 | 230 | L-Proline* | 0.027 | 1.75 |
|  | 21.11 | 246 | L-Cysteine* | 0.006 | 1.83 |
|  | 21.71 | 110 | Unknown 1 | 0.002 | 1.56 |
|  | 22.71 | 246 | L-Glutamate* | 0.000 | 2.3 |
|  | 22.74 | 192 | L-Phenylalanine* | 0.049 | 1.34 |
|  | 23.47 | 257 | FFA 12:0* | 0.004 | 1.86 |
|  | 24.89 | 217 | Arabitol* | 0.048 | 1.37 |
|  | 24.99 | 217 | Ribitol* | 0.009 | 1.38 |
|  | 30.58 | 333 | D-Gluconic acid* | 0.048 | 1.57 |
|  | 30.95 | 173 | Benzoate | 0.044 | 0.49 |
|  | 31.03 | 353 | Xanthine* | 0.022 | 0.58 |
|  | 32.44 | 437 | 2-Keto-d-gluconic acid | 0.013 | 0.67 |
|  | 32.58 | 456 | Urate* | 0.030 | 0.52 |
|  | 34.33 | 202 | 3-indolelactic acid | 0.003 | 0.62 |
|  | 35.50 | 232 | Unknown 2 | 0.046 | 1.24 |
|  | 36.83 | 70 | 2-Deoxy-D-ribose | 0.022 | 0.68 |
|  | 38.46 | 98 | N-alpha-acetyl-L-lysine* | 0.001 | 0.56 |
|  | 38.73 | 98 | FFA 18:0 | 0.037 | 0.67 |
|  | 40.26 | 91 | FFA 22:6* | 0.022 | 0.61 |
|  | 47.97 | 456 | 7-Hydroxycholesterol* | 0.004 | 2.24 |
|  | 7.95 | 117 | Lactate* | 0.036 | 1.2 |
|  | 8.67 | 147 | Pyruvate* | 0.017 | 1.28 |
| AT | 9.29 | 146 | Hydroxylamine* | 0.035 | 1.26 |
|  | 9.74 | 131 | 2-Hydroxybutyric acid* | 0.010 | 1.62 |
|  | 10.15 | 147 | Oxalate* | 0.021 | 0.79 |
|  | 10.71 | 233 | 3-Hydroxybutyric acid* | 0.007 | 1.86 |
|  | 12.08 | 131 | Hydroxyisovalericacid* | 0.043 | 0.77 |
|  | 12.41 | 73 | Glyceraldehyde* | 0.001 | 0.66 |
|  | 13.13 | 179 | Benzoate* | 0.001 | 1.38 |
|  | 13.79 | 158 | L-leucine* | 0.028 | 0.81 |
|  | 13.89 | 205 | Glycerol* | 0.001 | 1.8 |
|  | 14.37 | 158 | L-Isoleucine* | 0.022 | 0.79 |
|  | 14.39 | 130 | L-Threonine* | 0.036 | 0.78 |
|  | 14.44 | 142 | L-Proline* | 0.000 | 0.6 |
|  | 14.45 | 115 | Glutaric acid* | 0.001 | 0.58 |
|  | 15.41 | 189 | glycerate* | 0.008 | 0.81 |
|  | 18.66 | 248 | 3-Aminoisobutyrate* | 0.035 | 2.03 |
|  | 18.79 | 229 | FFA 10:0* | 0.003 | 1.66 |
|  | 20.31 | 232 | L-Aspartate* | 0.001 | 0.61 |
|  | 20.84 | 292 | L-Threonate* | 0.002 | 0.77 |
|  | 20.99 | 115 | Creatinine* | 0.001 | 0.66 |
|  | 21.11 | 246 | L-Cysteine* | 0.005 | 0.65 |
|  | 21.71 | 110 | Unknown 1 | 0.051 | 1.3 |
|  | 23.47 | 257 | FFA 12:0* | 0.021 | 1.52 |
|  | 23.81 | 116 | L-asparagine* | 0.016 | 0.57 |
|  | 25.83 | 292 | Ribonic acid | 0.006 | 0.67 |
|  | 25.85 | 357 | Glycerol 1-phosphate | 0.021 | 0.6 |
|  | 26.10 | 156 | L-glutamine* | 0.020 | 0.27 |
|  | 26.20 | 292 | 2-Keto-l-gluconic acid | 0.009 | 0.67 |
|  | 27.05 | 273 | Citric acid* | 0.008 | 1.7 |
|  | 27.12 | 245 | Isocitric acid* | 0.002 | 2 |
|  | 27.19 | 157 | Citrulline* | 0.017 | 0.65 |
|  | 27.47 | 283 | Myristoleicacid 14:1 | 0.004 | 2.51 |
|  | 27.73 | 285 | Myristic acid 14:0* | 0.001 | 1.76 |
|  | 29.72 | 299 | FFA 15:0* | 0.007 | 1.29 |
|  | 29.76 | 175 | Acetamide | 0.007 | 0.73 |
|  | 31.21 | 311 | Palmitelaidic acid FFA 16:1* | 0.004 | 2.11 |
|  | 31.63 | 313 | FFA 16:0* | 0.000 | 1.27 |
|  | 33.59 | 217 | Ribitol | 0.006 | 0.76 |
|  | 34.54 | 202 | L-Tryptophan* | 0.006 | 0.49 |
|  | 34.85 | 117 | FFA 18:1 | 0.001 | 1.54 |
|  | 35.22 | 117 | FFA 18:0* | 0.007 | 1.23 |
|  | 35.49 | 82 | FFA 18:2 | 0.002 | 1.54 |
|  | 35.50 | 232 | Unknown 2 | 0.018 | 0.78 |
|  | 35.82 | 232 | 2-Deoxy-D-ribose | 0.029 | 0.79 |
|  | 36.27 | 56 | 11,14-Eicosadienoic acid | 0.020 | 0.77 |
|  | 37.26 | 80 | FFA 20:4* | 0.007 | 0.71 |
|  | 37.37 | 158 | Xylonic acid | 0.018 | 0.73 |
|  | 38.09 | 367 | 11-Eicosenoic acid* | 0.003 | 1.52 |
|  | 49.14 | 202 | Leu-Trp | 0.012 | 0.59 |
